# Magnetic Microdevices for MRI-Based Detection of SARS-CoV-2 Viruses

**DOI:** 10.1109/OJEMB.2020.3026234

**Published:** 2020-09-24

**Authors:** Lamar O. Mair, Olivia Hale, Sahar Jafari, Cheng Chen, Oleg Udalov, Roland Probst, Ittai Baum, Anjana Hevaganinge, Elaine Yi Wang, Olga C. Rodriguez, Christopher Albanese, Stanley T. Fricke, Irving N. Weinberg

**Affiliations:** Neuroparticle Corporation North Bethesda MD 20852 USA; Shape Theranostics, Inc. and Weinberg Medical Physics, Inc. North Bethesda MD 20852 USA; Georgetown University 8368 Washington DC 20057 USA

**Keywords:** Coronavirus, COVID, magnetic microdevices, MRI, theranostic

## Abstract

*Goal:* To develop a micron-scale device that can operate as an MRI-based reporter for the presence of SARS-CoV-2 virus. *Methods:* Iron rod microdevices were constructed via template-guided synthesis and suspended in phosphate buffered saline (PBS). Heat-inactivated SARS-CoV-2 viruses were added to the samples and imaged with low-field MRI. *Results:* MRI of microdevices and viruses showed decreased signal intensity at low concentrations of viruses that recovered at higher concentrations. Electron micrographs suggest that reduced MRI intensity may be due to concentration-dependent shielding of water protons from local magnetic inhomogeneities caused by the iron microdevices. *Conclusions:* The preliminary results presented in this letter provide justification for further studies exploring the potential diagnostic role of magnetic microdevices in assessing the presence and concentration of SARS-CoV-2 viruses.

## I. Introduction

Imaging has long been used for guiding medical therapy [1]. Magnetic properties of iron particles have been used to speed processing of *in vitro* probes for quantifying SARS-CoV-2 viral load in biofluid samples [2], contributing to possible novel methods for faster, particle-based diagnostics. Magnetic nanoparticles have long been used as contrast agents in MRI [3]. Early experiments demonstrated the ability to detect proteins, biological processes, and viruses, with virus detection specificity and sensitivity below 1000 viruses per milliliter for herpes simplex virus [4]. The field of magnetic particle-based diagnostic magnetic resonance (DMR) has grown to include sensing and detection of numerous analytes, often with high specificity in low sample volumes [5]. Here we present preliminary results demonstrating the ability to rapidly image the SARS-CoV-2 virus in a solution of saline and iron microdevices using a low-field (0.34T) compact MRI. To our knowledge, these results represent the first application of magnetic microdevices combined with MRI for the characterization of SARS-CoV-2 virus [6], [7].

## II. Materials and Methods

Here we describe the magnetic microdevices used in our experiments, lay out the characteristics of the MRI hardware and software used in our acquisition process, and detail samples and sample preparation techniques.

### A. Magnetic Microdevices

Gold-tipped iron microdevices were synthesized in anodized aluminum oxide (AAO) templates via template guided electrodeposition following established protocols [8]–[10]. Briefly, AAO templates (Whatman Anodisc, nominal pore diameter 0.2 μm) were coated with 0.5 μm silver via thermal evaporation, forming a working electrode. AAO templates were then placed in a custom electroplating cell and gold, iron, and gold layers were plated by sequential deposition using DC potentials. Following plating, the silver working electrode was dissolved in nitric acid and the AAO template material was dissolved in 1 M NaOH, releasing the magnetic microdevices into solution. Microwires were rinsed in ethanol and, prior to experiments, suspended in phosphate buffered saline (PBS). Microdevices in wire configurations (270 nm in diameter and 7.5 μm in length) were prepared in batches of one billion particles. In separate short-term *in vivo* toxicity experiments, approved by the Georgetown University Institutional Animal Care and Use Committee (IACUC Protocol #2019-0048), particles were instilled into the right nares of six female C57BL6 mice (4-8 months of age). Animals were euthanized four hours later.

### B. Samples and Sample Preparation

SARS-CoV-2 capsids were obtained from BEI Resources (NR-52286, Manassas, Virginia, USA). The SARS-CoV-2 sample is an isolate of USA-WA1/2020 having 3.75 × 10^8^ copies/ml. Six samples were prepared (A-F) and consisted of combinations of PBS, microdevices, and SARS-CoV-2 capsids in various concentrations. Sample components were mixed in 50 μl of PBS (total sample volume) and inserted into borosilicate glass culture tubes (4.5 mm inner diameter). Samples were sealed with parafilm, then loaded into an RF coil for MR imaging experiments. A control sample of PBS only (sample A), as well as a microdevice reference sample containing 2 × 10^6^ microdevices in PBS was generated (sample B). Two viral concentrations (7.5 × 10^7^ and 30 × 10^7^ copies/ml) were tested with 2 × 10^6^ microwires in each test sample (samples C and D, respectively). Test samples were compared with control PBS (sample A), reference sample of PBS with microdevices (sample B), and further control samples of PBS and virus (samples E and F, viral concentrations of 7.5 × 10^7^ and 30 × 10^7^ copies/ml respectively). Sample contents are summarized in Table I.

**TABLE I:** 

	A	B	C	D	E	F
PBS [μl]	50	50	50	50	50	50
microwires [ × 10^6^]	0	2	2	2	0	0
SARS-CoV-2 [ × 10^7^ copies/ml]	0	0	7.5	30	7.5	30

### C. Magnetic Resonance Imaging of the Samples

MR imaging experiments were performed using a 0.34T (14.5 MHz) NdFeB permanent magnet MRI (SpinCore Technologies, Gainesville, Florida USA). RF excitation and acquisition were performed using a portable SpinCore iSpin-NMR console. Pulse sequence generation was performed using GNU Radio MRI (gr-MRI) based on previous work by Hasselwander et al. [11] Spin echo sequences with a repetition time of TR = 4 s and an echo time of TE = 50 ms were used for excitation. Data was collected, stored, then converted to images via 2D inverse FFT reconstruction. Collected images were imported to ImageJ. Rectangular regions-of-interest were drawn over the centers of the projection images and analyzed using ImageJ tools for determining average and standard deviations of mean intensity. Following MRI experiments, samples were deposited onto silicon wafers and imaged via scanning electron microscopy (Phenom XL, Nanoimages LLC, Pleasanton, CA).

## III. Results

### A. Virus MR Imaging Studies

**Fig. 1.**
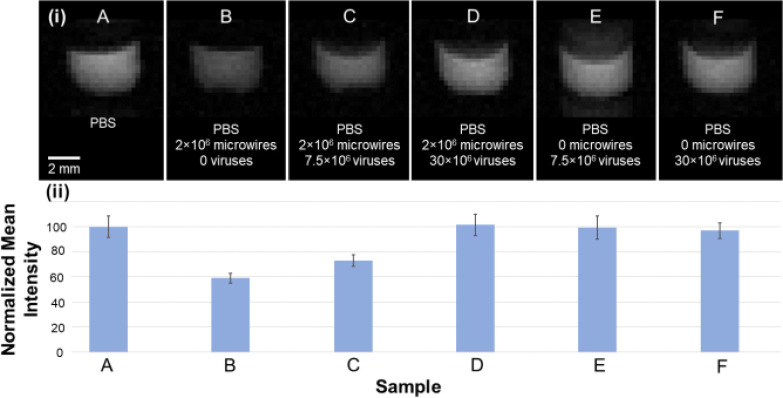
(i) MR images of 50 μl PBS samples with various concentrations of micro-devices. (A) No microdevices, no viruses. (B) Low intensity seen after adding 2 × 10^6^ micro-devices, no viruses. (C) Reduced intensity with 2 × 10^6^ micro-devices, 7.5 × 10^7^ copies/ml. (D) Recovery of intensity with 2 × 10^6^ micro-devices, 30 × 10^7^ copies/ml. Control images on samples having only viruses (E, F) suggest that viral content alone, at the concentrations shown, is insufficient to appreciably alter mean intensity.

 MR images of all samples containing microwires show significant signal reduction caused by the presence of the microdevices at the base of the sample vial (Fig. 1(i), B-C). Additionally, samples B and C show a diminished overall intensity in the region of interest. The presence of SARS-CoV-2 virus in PBS alone does not alter the overall average signal intensity when microdevices are not present (Fig. 1(ii), E-F).

### B. Virus SEM Imaging Studies

**Fig. 2.**
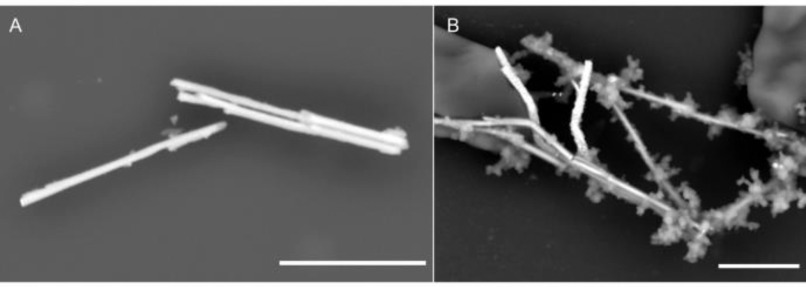
(A) Microdevices extracted from sample containing only PBS and microwires. As expected, wire surface is smooth and contains no extraneous material or nonspecifically bound material. (B) Microdevices extracted from sample containing PBS, SARS-CoV-2 viruses, and microwires. Microdevices appear coated with viral capsids. Both scale bars are 5 μm.

To inspect microdevices in PBS without viruses as compared with microdevices in virus-laden PBS samples, we imaged them under scanning electron microscopy (SEM). While microdevices extracted from PBS-only samples appeared smooth (Fig. 2A), microdevices which been mixed with virus capsids were coated with adsorbed virus material (Fig. 2B).

### C. Immediate Reaction Studies in Mice

None of the mice demonstrated immediate reactions to the inhaled microdevices.

## IV. Discussion

Ferromagnetic micromaterials provide contrast in MRI by creating local magnetic inhomogeneities that affect the spin dynamics of nearby protons [12]. Modification of proton spin dynamics in the vicinity of shape-tuned and shape-changing magnetic microdevices has been used to generate MRI contrast [13], [14]. Our SEM studies (Fig. 2) demonstrate that in the presence of SARS-CoV-2 capsids, the iron microdevices become coated with viruses, and it is therefore reasonable to assume that this coating and the attendant separation of protons from the microdevices are responsible for the contrast we observe. Here we demonstrate detection of 7.5 × 10^7^ copies/ml. Previous studies have reported ranges between 10^2^ and 10^9^ copies/ml in sputum samples from infected individuals [15]. Further experiments probing the lower limits of detection, the significance of microdevice dimensions and concentration, and the time-dependence of signal recovery will inform how microdevices interact with SARS-CoV-2 virus capsids to alter MR signal intensity and what limits of detection may be obtainable with the technique. Follow-on experiments in more complex media, such as blood and cell suspensions, will also be useful in determining how the described technique may operate in complex samples. The binding of viruses to the microdevices might be more selectively accomplished with specific microdevice shapes, chemo-attractants, or surface functionalization [16]. As the effect is dependent upon microdevice concentration, increasing microdevice concentration might increase the upper limit of detection SARS-CoV-2 viruses.

In experiments using similar rod-like microdevices, Poland *et al*. demonstrated that microdevice length is a significant factor in pathogenesis and toxicity, noting that microwires less than 5 μm long induced no observable inflammation or fibrosis when injected into the peritoneum. Those studies demonstrated significant granulomas and inflammation for 20 μm long microwires in the lungs, but showed little effects (beyond mild alveolar inflammation) for 5 μm long microrods [17]. Additional testing in other organs and systems of interest to the COVID-19 community (e.g. lungs, pharynx, kidneys, heart) will be required to investigate the safety and effectiveness of the microdevices before proceeding to human testing.

The results are intriguing with respect to the possibility of using the microdevices as theranostics. Chemical methods of destroying capsids have been proposed as therapeutic modalities [18]. The ability to mechanically damage viral capsids has been studied in terms of burst-pressure tolerance [19]. The described microdevices have previously been used for disruption of microbial biofilms adhered to surfaces [20], and future experiments will explore the possible theranostic applications of such microdevices by combining imaging and micro-mechanical disruption applied to the virus capsids. Magnetic transport of the microdevices described in this report [21] may enable targeted transport to specific tissues. The described microdevices have been implanted within rat brain tissues *in vivo*, with no ill effects observed after one month [22].

Exploitation of the theranostic application might require higher magnetic gradients than are usually available on conventional MRI instruments, but could potentially be accommodated with custom devices [23], [24].

## V. Conclusion

We have shown that ferromagnetic microdevices can be used as contrast agents for SARS-CoV-2 viruses in PBS using MR imaging, with results available in minutes. Potential theranostic applications of such microdevices are intriguing possibilities.
